# The candidate oncogene (MCRS1) promotes the growth of human lung cancer cells via the miR–155–Rb1 pathway

**DOI:** 10.1186/s13046-015-0235-5

**Published:** 2015-10-14

**Authors:** Minxia Liu, Kecheng Zhou, Yunchao Huang, Yi Cao

**Affiliations:** Laboratory of Molecular and Experimental Pathology, Kunming Institute of Zoology, Chinese Academy of Sciences, Kunming, China; Kunming College of Life Science, University of Chinese Academy of Sciences, Kunming, China; Department of Thoracic and Cardiovascular Surgery, The Third Affiliated Hospital of Kunming Medical University (Yunnan Tumor Hospital), Kunming, China

**Keywords:** Lung cancer, MCRS1, miR-155, Rb1, Proliferation, DNA copy number

## Abstract

**Background:**

Microspherule protein 1 (MCRS1) is a candidate oncogene and participates in various cellular processes, including growth, migration, senescence and transformation. MCRS1 is overexpressed in non-small cell lung cancer (NSCLC) and promotes the growth of cancer cells. However, the mechanisms driving these processes are not fully understood.

**Methods:**

Retrovirus-mediated RNA interference was employed to knockdown MCRS1 expression in cell lines. Cell proliferation assays and animal experiments were respectively performed to evaluate the growth of NSCLC cells *in vitro and in vivo.* Microarray analysis was carried out for mRNA profiling. Luciferase reporter assay and microRNA (miRNA) transfection were used to investigate the interaction between miRNA and gene.

**Results:**

Stably knocking down MCRS1 expression inhibited the proliferation of NSCLC cells *in vitro* and *in vivo*. By comparing the mRNA expression profiles of NSCLC cells with or without MCRS1 silencing, we found that MCRS1 regulated expressions of various genes related to cell proliferation, including Rb1, TP53, cell cycle-related genes, MYC, E2F2, PCNA, and Ki67. However, MCRS1 did not directly bind to these differentially expressed genes. Here, we confirmed that Rb1, an important tumor suppression gene (TSG), is a direct target of miR-155 which is directly up-regulated by MCRS1. Furthermore, the level of Rb1 expression in NSCLC tissues was inversely correlated with those of miR-155 and MCRS1, and MCRS1 regulated expression of Rb1 via miR-155. Additionally, we found that the DNA copy number of the MCRS1 gene played a role in MCRS1 overexpression in NSCLCs.

**Conclusion:**

MCRS1 overexpression induced NSCLC proliferation through the miR-155–Rb1 pathway and DNA copy-number amplification is one of the mechanisms underlying MCRS1 overexpression in NSCLC. Moreover, we put forward the hypothesis that there are regulatory relationships between oncogenes and TSGs apart from the functional synergy of both; the oncogene-miRNA-TSG networks are one of mechanisms among the regulatory relationships; the regulatory relationships and the networks might play active roles in the development and progression of cancer.

**Electronic supplementary material:**

The online version of this article (doi:10.1186/s13046-015-0235-5) contains supplementary material, which is available to authorized users.

## Background

Lung cancer is the leading cause of cancer-related death in the world. Lung cancer can be divided into small-cell lung cancers and non-small cell lung cancers (NSCLCs), the latter of which account for more than 80 % of all types of lung cancer, including adenocarcinomas (ACs), squamous cell carcinomas (SCCs) and large-cell carcinomas (LCCs). Lung cancer development is a complex process involving the accumulation of genetic and epigenetic alterations, resulting in uncontrolled proliferation, cellular transformation and the acquisition of aggressive behavior. Although much effort has been invested in identifying the genes related to lung cancer, the mechanisms underlying this disease are not fully understood.

In previous studies, we screened novel lung cancer-related genes using a combination of cytogenetic and molecular genetic methods and found that microspherule protein 1 (MCRS1) was overexpressed in NSCLCs [1, 2]. Furthermore, abnormal expression of MCRS1 was found to be correlated with cellular proliferation, the epithelial-mesenchymal transition (EMT) and metastasis, implying that MCRS1 may play critical roles in lung tumorigenesis [2, 3]. MCRS1, also known as p78 and MSP58, was initially identified as an interacting partner of the proliferation-related nucleolar protein p120 and was localized to the nucleolus [4]. Consistent with its nucleolar location, MCRS1 was found to mediate distinct cellular functions through interactions with various proteins present in this organelle, including nucleolar proteins, transcription factors and RNA-binding proteins [4–9]. In previous studies, a small fraction of p78/MCRS1 was found to co-localize with centrosomal proteins, such as Nde1 and DIPA, suggesting that MCRS1 plays a role in centrosomal homeostasis [10, 11]. Notably, MCRS1 exhibited transformative properties in fibroblast cells, whereas the PTEN tumor suppressor inhibited this transformative property [12, 13]. Moreover, MCRS1 silencing was reported to suppress the proliferation, migration and invasion of the tumor cells of other types of cancers [14–17]. Based on the transformative properties of MCRS1 and its biological roles in various types of human cancers, MCRS1 can be considered a candidate oncogene. However, the mechanism(s) underlying the enhanced expression of MCRS1 and the consequences of its aberrant regulation are not fully understood. In the present study, we investigated the effect of MCRS1 on the proliferation of NSCLC cells *in vivo* and *in vitro*, as well as the mechanisms driving this process.

## Materials and methods

### Ethics statement

NSCLC and adjacent non-tumor lung tissues were obtained from patients during surgery in Kunming Medical University. The study was approved by the Ethics Committee for Human Medicine Research, Kunming Institute of Zoology, Chinese Academy of Sciences (Permit Number: SYDW-2012010). Written informed consent was obtained from the patients before obtaining samples for this study.

The animal experiments were carried out in strict accordance with the guidelines and approved by the Ethics Committee for Animal Experimentation, Kunming Institute of Zoology (Permit Number: SYDW-2012010).

### NSCLC tissue samples and cell lines

Samples of primary NSCLC tissues were obtained from 30 patients during surgery. None of the patients had been treated before undergoing surgery. All of these samples were diagnosed according to the classification system of the World Health Organization and were staged using the criteria of the International Union Against Cancer [18, 19]. Samples of ten lung tissues, which were obtained from patients with lung bullous disease and lung inflammatory pseudotumors who were undergoing surgery, were used as the “noncancerous tissues.” The clinical characteristics of patients are listed in Additional file 1.

The lung cancer cell lines EPLC-32M1 (SCC), A549 (AC), 801D (LCC), NCIH-292 (mucoepidermoid carcinoma), NCI-H460 (LCC) and 16HBE (immortalized human bronchial epithelial cells) have been described previously [2, 3]. And details of all cell lines were provided in Additional file 2.

### Generation of stable subcellular MCRS1 knockdown lines

The MCRS1 silencing vector was previously described [2]. A target sequence (GCTGAAGAACAACGGTGAT) was designed and produced for MCRS1 silencing, and a luciferase-targeting shRNA oligonucleotide was used as the negative control. The complementary strands of the oligonucleotides were annealed and were ligated into the prelinearized RNAi-Ready pSIREN-RetroQ vector (Clontech, Palo Alto, CA, USA). The MCRS1-silencing and negative control vectors were transfected into RetroPack PT-67 cells using Lipofectamine 2000 reagent (Invitrogen, Carlsbad, CA, USA). Forty-eight hours after transfection, the retrovirus-containing medium was filtered, and the retroviruses were used to infect the cells of NSCLC lines in the presence of 10 μg/ml Polybrene (Sigma, St. Louis, MO, USA). Forty-eight hours after infection, the cells were selected using puromycin (Sigma).

### Oligonucleotide transfection

The miR-155 mimics, 2′-O-methylated miRNA antisense oligonucleotides (ASO) and their cognate controls that were used for transient transfection, were purchased from the RiBoBio Company (Guangzhou, China). Transfection was performed using Lipofectamine 2000 (Invitrogen) according to the manufacturer’s instructions. In each case, 100 nmol/L of the miRNA mimic and 150 nmol/L of the ASO were used.

### Vector construction

To generate the Rb1 (retinoblastoma-related gene 1) luciferase reporter plasmid (pGL-Rb1), the 3′ untranslated region (3′-UTR) encompassing the miR-155 binding site was cloned between the *Kpn*I and *Xho*I restriction sites of the pGL-3 basic vector (Promega, Madison, WI, USA) using a Polymerase Chain Reaction (PCR)-generated fragment. The mutation in the miR-155 binding site of the human Rb1 3′-UTR was generated using overlapping PCR. For the ectopic expression of Rb1, the coding region of human Rb1 was cloned into the pCDH-CMV-MCS-EF1-GFP-T2A-Puro vector (System Biosciences, California, USA). Subsequently, virus packaging and infection were performed according to manufacturer’s instructions. The contents of all of these constructs were confirmed by sequencing. The primers used are shown in Additional file 3.

### MiRNA target prediction and luciferase reporter assays

The miRWalk database was used to predict the target genes of miRNAs [20]. The genes that had both downregulated expression after MCRS1 silencing and had been predicted to be targets of miR-155 were chosen for analysis using the reporter assay.

Each reporter construct and the *Renilla* luciferase expression plasmid (pRL-TK) was co-transfected into cells with the miR-155 mimic or its negative control using Lipofectamine 2000. The pRL-TK plasmid was used as an internal control. Forty-eight hours after transfection, the levels of luciferase activity were determined using the Dual-Luciferase Reporter System (Promega) according to the manufacturer’s instructions.

### Quantitative real-time polymerase chain reaction (QRT-PCR) assays of miRNA and mRNA expression

The total RNA was extracted from cells and tissues using TRIzol reagent (Sigma). The qRT-PCR assays of mRNA and miRNA expression levels were conducted as previously described [3]. The housekeeping genes GAPDH and U6 snRNA were used as internal controls for the mRNA and miRNA assays, respectively. The primers used are shown in Additional file 3.

### Western blotting analysis

The preparation of protein-containing lysates and western blotting was conducted as described previously [3]. Briefly, the proteins in the lysates were resolved using 10 % sodium dodecylsulfate polyacrylamide gel electrophoresis (SDS-PAGE) and were transferred to PVDF membranes (Millipore, Bedford, MA, USA). The membranes were incubated with the following antibodies: anti-Rb1 (sc-50; Santa Cruz Biotechnology Inc., Santa Cruz, CA, USA), anti-MCRS1 (R36649; Sigma), and anti-GAPDH (G8795; Sigma). Finally, the membrane was incubated with SuperSignal West Pico chemiluminescent substrate (Thermo Fisher Scientific Inc., Waltham, MA, USA), and the blots were exposed to X-ray film. The films were developed, scanned and analyzed using the Image J software (NIH, Bethesda, MD, USA). GAPDH was used as an internal control.

### MTT assay

Cells were seeded at a density of 4 × 10^3^ cells/well in 96-well plates. Seventy-two hours post-transfection, MTT (3-(4,5-dimethylthiazol-2-yl)-2,5-diphenyltetrazolium bromide) was added to each well. After an incubation period, the medium was removed, and dimethyl sulfoxide (DMSO) was added. The absorbance at 570 nm (using 630 nm as a reference) was detected using a microplate reader (model 680, Bio-Rad Laboratories, Berkeley, CA, USA).

### *In vivo* tumor growth assay

Sixteen female BALB/c nude mice (4 weeks old) were purchased from Vital River Laboratories (Beijing, China) and were housed under standard conditions. Overall, 1 × 10^6^ control EPLC-32 M1 cells (without MCRS1 silencing) and MCRS1-knockdown EPLC-32 M1 cells were subcutaneously implanted into the left and right flanks of the same mouse, respectively. Tumor growth was assessed using calipers every five days from 4 days to 32 days post-implantation, and the tumor volumes were estimated using the following equation: 0.5 × length × width^2^. The mice were anesthetized with diethyl ether and sacrificed by cervical dislocation at 5 weeks post-implantation, and the tumor pairs were harvested and weighed.

### cDNA microarray analysis

The total RNA was extracted from MCRS1 knockdowned cells and control cells using TRIzol reagent (Sigma). Agilent 60 K Human Gene Expression arrays were used for mRNA profiling. Quality control of the total RNA, the probe labeling, and the array hybridization, as well as the data extraction and normalization, were performed at the CapitalBio Corporation (Beijing, China; http://www.capitalbio.com). The differentially expressed genes were determined according to the ratio of their expression levels relative to those of the control cells (ratio > 2: upregulated genes; ratio < 0.5: downregulated genes). The differentially expressed genes are shown in Additional file 4.

### Chromatin immunoprecipitation (ChIP) assays

The ChIP assays were performed using the EZ-Magna ChIP kit (Millipore, Merck KGaA, Darmstadt, Germany) as described previously [3]. Briefly, the chromatin of the samples was sheared into fragments with an average length of approximately 250 bp by sonication on ice using a Bioruptor sonicator. Immunoprecipitation of the sonicated chromatin fragments was conducted using an anti-MCRS1 antibody (SC-376569; Santa Cruz Biotechnology) and normal mouse IgG antibodies (the negative control), and the input and immunoprecipitated DNA fragments were analyzed using PCR. The GAPDH promoter primers provided in the ChIP kit were used as the internal control. The primers used are shown in Additional file 3.

### DNA copy number assays

Genomic DNA was extracted from tissues and cultured cells using standard methods. After digesting the samples using proteinase K, the genomic DNA was isolated using phenol/chloroform/isoamyl alcohol and ethanol precipitation. Then, based on the instructions of the qBiomarker Copy Number PCR assay kit, each sample DNA was diluted to the same concentration and was amplified using qRT-PCR. The results were analyzed using the qBiomarker Data Analysis program (http://pcrdataanalysis.sabiosciences.com/cnv/CNVanalysis.php). The DNA copy number was determined using the 2^-△CT^ method. The primer set used to determine the MCRS1 copy number was as follows: 5′CACCAGAAGGCTCACTCTTCA3′ and 5′TGTCTGGTTGTCGAAGTCCGG3′. The qBiomarker Multicopy Reference Assay (*MRef,* QIAGEN, Suzhou, China) was used as the internal control.

## Statistical analysis

The data are presented as the mean values ± standard deviation (SD) as obtained from at least three independent experiments. The data were analyzed using the SPSS (Statistical Package for the Social Sciences) 17.0 software package (Chicago, IL, USA). The obtained data (levels of mRNA, the results of the MTT assay the *in vivo* tumor growth assay and the DNA copy numbers) were analyzed using Student’s *t*-test or a one-way ANOVA, as appropriate. The relationships among the levels of miRNA expression, alterations in the copy number and the levels of gene expression were evaluated using Pearson’s correlation coefficients. The level of significance was set at 0.05 for all tests.

## Results

### Reducing the level of MCRS1 attenuates the growth of NSCLC cells *in vitro* and *in vivo*

**Fig. 1:**
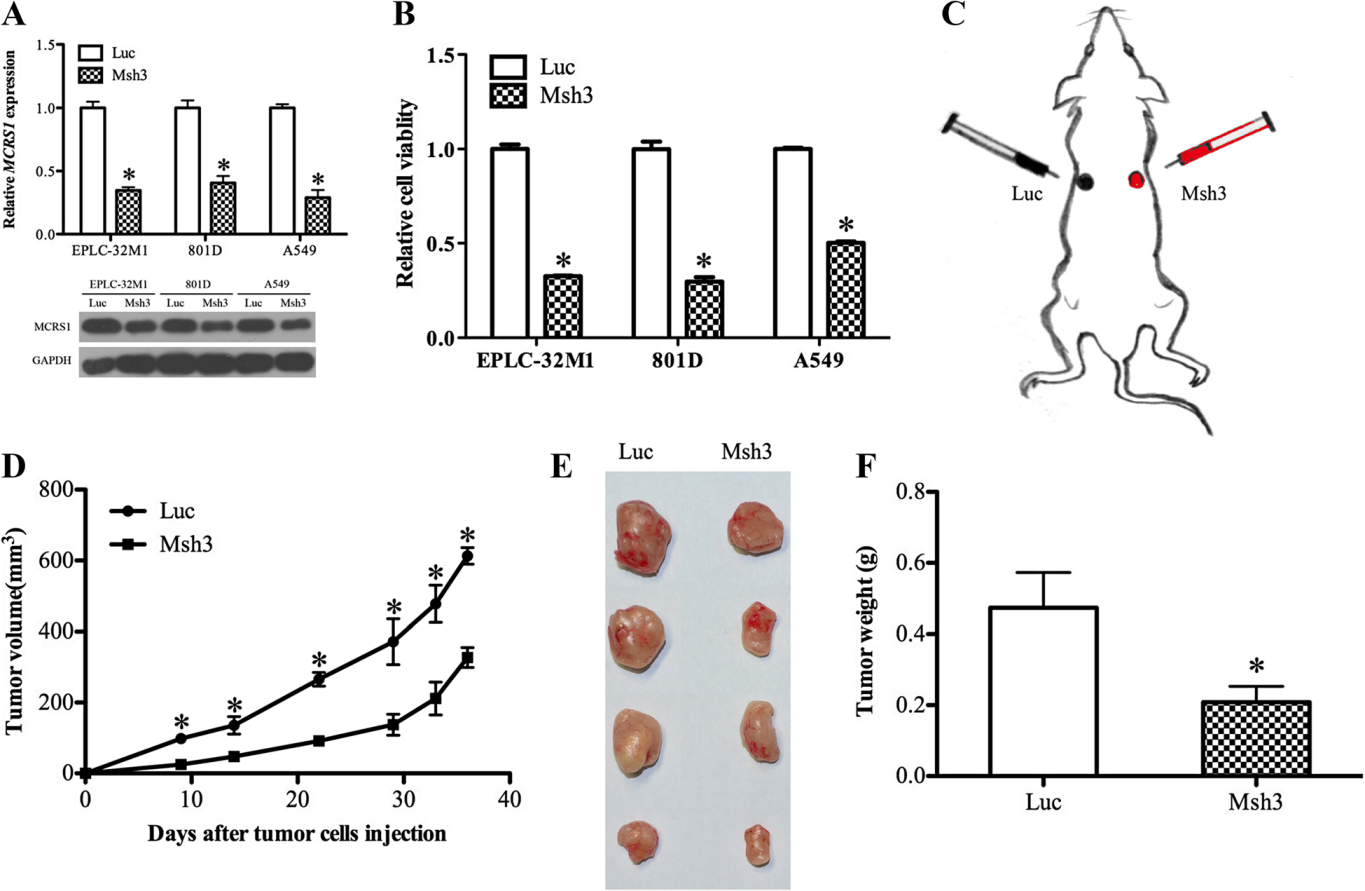
Stably knocking down the expression of MCRS1 inhibited the proliferation of NSCLC cells *in vivo* and *in vitro*. **a** The stable knockdown of MCRS1 expression decreased the levels of MCRS1 mRNA and protein in NSCLC cells compared with those of control cells without the MCRS1 knockdown. **b** The stable knockdown of MCRS1 expression notably reduced the level of cell viability, as evaluated using an MTT assay. **c** A diagram of EPLC-32 M1 cells with stably reduced MCRS1 expression and the control cells that were subcutaneously implanted into nude mice. **d** The stable knockdown of MCRS1 expression significantly suppressed tumor growth in nude mice. The differences between MCRS1 knockdown cells and the matched control cells were analyzed by Student’s *t*-test (*P* < 0.05). **e** Representative images of tumors at five weeks following the subcutaneous implantation of EPLC-32 M1 cells with or without MCRS1 knockdown in nude mice. **f** The stable knockdown of MCRS1 expression dramatically decreased the tumor weight relative to that of tumors derived from control cells. Msh3: cells with knocked-down MCRS1 expression; Luc: cells without knocked-down MCRS1 expression. *P < 0.05 (Student’s *t*-test)

To investigate the effect of MCRS1 on tumor formation, we stably reduced the level of MCRS1 expression using RNA interference mediated by a retroviral system (pSIREN-RetroQ) in EPLC-32 M1, A549 and 801D cells, representing SCC, AC and LCC cells, respectively. The decreased level of MCRS1 expression was assessed using qRT-PCR and western blotting assays (Fig. 1a).

The results of the cell proliferation assays showed that MCRS1 silencing significantly decreased the rate of NSCLC tumor cell proliferation *in vitro* (Fig. 1b). To further study the effect of MCRS1 silencing on NSCLC tumor formation *in vivo*, MCRS1-knockdown cells (Msh3) and control cells (Luc) were subcutaneously injected into the opposite flanks of nude mice (Fig. 1c). Compared with the tumors derived from the control cells, the tumors derived from the Msh3 cells displayed a significantly smaller size and mass (Figs. 1d-f). Collectively, the *in vitro* and *in vivo* analyses supported the suppressive effect of MCRS1 silencing on tumor cell growth, indicating that MCRS1 expression may play a role in the growth of NSCLC cells.

### MCRS1 knockdown induces the differential expression of genes related to cellular proliferation

**Fig. 2:**
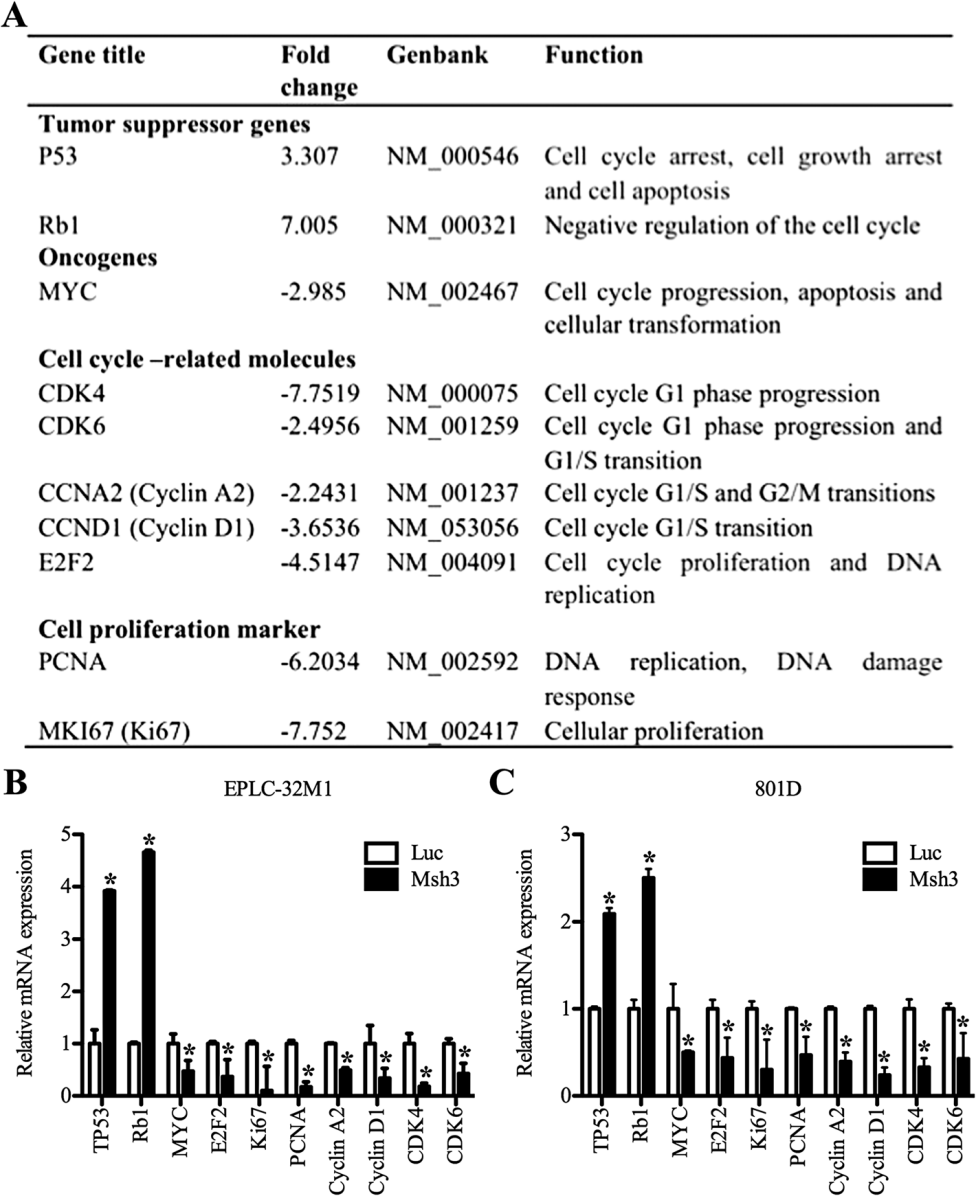
Genes related to cell proliferation that were differentially expressed upon knocked-down MCRS1 expression. **a** The results of an mRNA microarray analysis of differentially expressed genes related to cell proliferation in EPLC-32 M1 cells in which the expression of MCRS1 was knocked down. +, Upregulated; -, Downregulated. **b** and **c** qRT-PCR validation of the genes that were differentially expressed in EPLC-32 M1 and 801D cells with (Msh3 cells) and without (Luc cells) the knocked-down expression of MCRS1. The results were analyzed using Student’s *t*-test (*P* < 0.05)

To explore the molecular mechanism(s) underlying MCRS1-mediated cellular growth, we utilized a cDNA microarray to determine the mRNA profiles of EPLC-32 M1 cells with knocked-down MCRS1 expression. The array data revealed that the expression levels of 369 genes were significantly altered after MCRS1 silencing. Notably, among these altered genes, the levels of expression of the MYC oncogene and the genes for the proliferation factor E2F2 and the proliferation markers Ki67 and PCNA were significantly downregulated. In contrast, the levels of expression of the tumor suppressor genes (TSG) TP53 and Rb1 were upregulated. Additionally, the levels of expression of cell cycle-related genes, such as Cyclin A2, Cyclin D1 and CDK4/6, were markedly changed (Fig. 2a). Subsequently, a qRT-PCR assay was used to verify the levels of expressions of the genes listed in Fig. 2a. Although the exact fold changes in the levels of expression of these genes varied somewhat between the data from the array and qRT-PCR, the data displayed similar trends (Fig. 2b and c).

Considering that MCRS1 is involved in distinct cellular processes through activating various proteins, we further sought to determine whether MCRS1 bound to these genes, as shown in Fig. 2b and c. Regrettably, the predictions obtained using the transcription factor binding site and promoter database suggested that the promoter regions of these gene were not targets of MCRS1, indicating that MCRS1 may regulate these genes via other pathways, such as microRNA (miRNA)-based pathways.

Taken together, these results indicated that MCRS1 could affect tumor cell growth through indirectly modulating the expression of pro-proliferative, anti-proliferative and cell cycle-related genes.

### MCRS1 regulates cellular growth via miR-155 targeting of the Rb1 gene

**Fig. 3:**
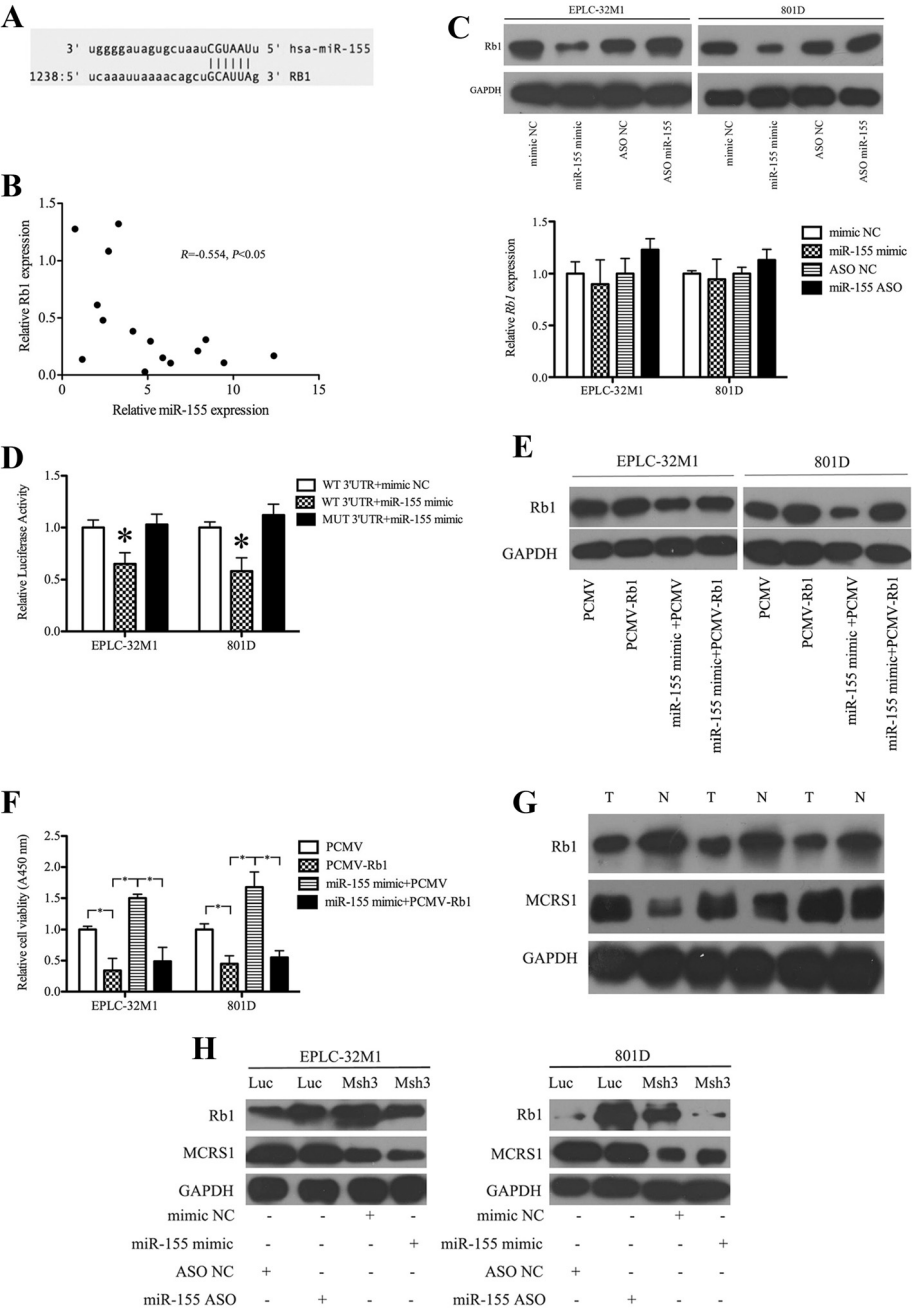
MCRS1 regulated cellular proliferation via miR-155 targeting Rb1 mRNA in NSCLCs. **a** Schematic of the putative miR-155 binding site in the Rb1 3′UTR region. **b** The results of the correlation analysis of the levels of miR-155 and Rb1 protein in NSCLC tissues (Pearson’s method, *R* =−0.554, *P < 0.05). **c** Relative levels of expression of Rb1 protein (top) and Rb1 mRNA (bottom) in cells transfected with the miR-155 mimic, the NC mimic, an miR-155 inhibitor (miR-155 ASO) or NC ASO, respectively. (Student’s *t*-test, *P < 0.05). **d** Validation of the direct targeting of Rb1 mRNA by miR-155 was obtained using a luciferase reporter assay. WT: wild-type Rb1 3′UTR; MUT: mutant Rb1 3′UTR. (Student’s *t*-test, *P < 0.05). **e** The inhibition of Rb1 expression by miR-155 was partially reversed by co-transfection with pCMV-Rb1 (to drive the ectopic expression of Rb1 mRNA lacking the 3′UTR to which miR-155 bound) and the miR-155 mimic. **f** The level of cell viability was reduced by transfection with PCMV-Rb1. Importantly, the induction of a higher level of cell viability by the miR-155 mimic was partially prevented by PCMV-Rb1 transfection. **g** The relative levels of expression of Rb1 and MCRS1 protein in clinical samples. T: Tumor; N: Noncancerous. **h** The lower level of Rb1 protein in cancer cells without MCRS1 silencing (Luc) was increased by treatment with the miR-155 inhibitor, and the higher level of Rb1 protein in the MCRS1-knockdown cells (Msh3) was decreased by treatment with the miR-155 mimic. NC: negative control. ASO: antisense oligonucleotide

Our previous study demonstrated that miR-155 is a downstream node of MCRS1 in lung cancer cells [3]. MCRS1 has been reported to regulate cellular proliferation and the cell cycle [16, 21, 22]. Based on this background, we attempted to determine whether MCRS1 modulates the proliferation of NSCLC cells via miR-155-targeted genes. An integrated analysis of miR-155 target prediction and verification of the microarray data using qRT-PCR (Fig. 2b and c) indicated that the genes encoding Rb1 and E2F2 might be targeted by miR-155. Rb1 was chosen for further studies based on the following two criteria: (1) Rb1 was predicted to be a target gene of miR-155 (Fig. 3a) and plays a critical role in cellular proliferation [23, 24]; and (2) Rb1 protein levels were negatively correlated with miR-155 levels in lung cancer tissues (Fig. 3b).

The forced expression of miR-155 greatly reduced the level of Rb1 protein, and conversely, inhibiting miR-155 expression enhanced Rb1 protein expression in EPLC-32 M1 and 801D cells (Fig. 3c, top panel), although the Rb1 mRNA levels were not significantly different (Fig. 3c, bottom). A luciferase reporter assay conducted using reporter gene constructs that contained the Rb1 3′ UTR showed that the luminescence intensities were significantly reduced following the addition of an miR-155 mimic. Furthermore, mutation of the putative miR-155 binding site in the Rb1 gene abrogated the repression of luciferase activity caused by miR-155 overexpression (Fig. 3d). To further confirm that the growth-promoting effect of miR-155 was mediated by repressing Rb1 expression, a pCMV-Rb vector containing only the Rb1 coding sequence was constructed for the expression of Rb1. As shown in Fig. 3e, the level of Rb1 was restored after its exogenous expression via pCMV-Rb1. Additionally, Rb1 re-expression effectively suppressed the promotion of cellular proliferation caused by miR-155 overexpression (Fig. 3f). These results demonstrated that Rb1 is a functional target of miR-155 in NSCLC cells.

Based on our previous study showing that miR-155 is a downstream mediator of the function of MCRS1 in cellular proliferation and the EMT process [3], we examined whether MCRS1 regulated tumor cell growth through miR-155 targeting of the Rb1 gene. As shown in Fig. 3g, Rb1 protein expression was downregulated in MCRS1-overexpressing NSCLC tissues. In addition, the ectopic expression of miR-155 abrogated the increased expression of Rb1 protein in NSCLC cells with stably reduced MCRS1 expression (Msh3 cells), whereas inhibiting miR-155 expression partially restored the higher Rb1 level in NSCLC control cells without MCRS1 silencing (Luc cells) (Fig. 3h). In NSCLC samples, MCRS1 protein expression showed positive and inversely correlation with miR-155 expression and Rb1 protein expression, respectively (Additional file 5). Collectively, these data confirmed that MCRS1 promotes the proliferation of NSCLC cells via miR-155 targeting of the Rb1 gene.

### MCRS1 overexpression is partially caused by alterations in gene copy number

**Fig. 4:**
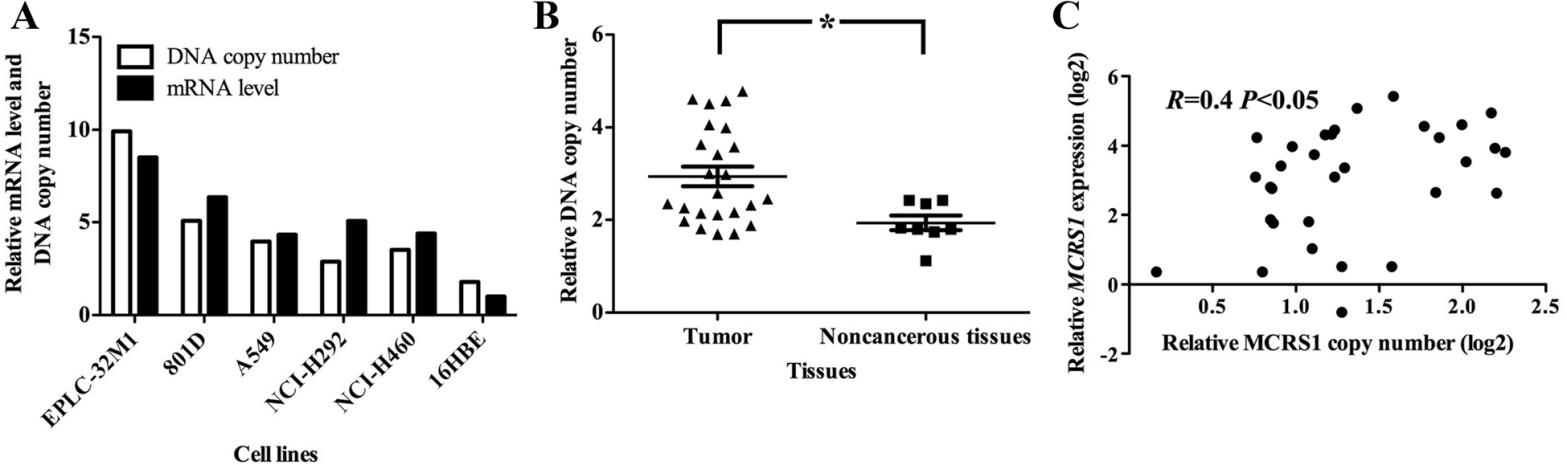
MCRS1 DNA copy numbers and MCRS1 mRNA expression levels. **a** Changes in the MCRS1 gene copy number and the level of MCRS1 mRNA expression in cultured cells were evaluated using qRT-PCR. **b** The variation in the MCRS1 DNA copy number in the NSCLC tissues was compared with that of noncancerous lung tissues. (Student’s *t*-test, *P < 0.05). **c** The results of the correlation analysis of the MCRS1 mRNA expression levels and the MCRS1 copy number alterations. (Pearson’s method, *R* = 0.4, *P < 0.05)

Because alterations in the DNA copy number have a dramatic impact on the level of gene expression, we next assessed whether changes in the MCRS1 DNA copy number contributed to the overexpression of MCRS1. As shown in Fig. 4a, the DNA copy number and the level of mRNA expression of MCRS1 were markedly elevated in each cancer cell line compared with those of the 16HBE cell line. Furthermore, the average DNA copy number of MCRS1 was increased by approximately 1.5-fold in the NSCLC tissues compared with that of the noncancerous lung tissues (Fig. 4b). The statistical analysis revealed that the DNA copy number was correlated with the level of mRNA expression of MCRS1 (Fig. 4c). Moreover, statistical analysis showed there are positive correlations between MCRS1 protein expression and MCRS1 copy number, as well as MCRS1 protein expression and its mRNA expression in NSCLC tissues (Additional file 6). These data indicated that, at least in part, the MCRS1 gene copy number affected the expression levels of MCRS1 mRNA and protein.

## Discussion

MCRS1 has been reported to be associated with various functions, such as transcriptional regulation and telomerase inhibition and to be involved in cellular growth, migration, senescence and transformation [5, 8, 9, 12, 13, 21, 22, 25]. Mounting evidence has demonstrated that MCRS1 is overexpressed in human esophageal carcinomas, hepatocellular carcinomas and colorectal carcinomas [14–16]. In previous studies, we showed that MCRS1 is abundantly expressed in NSCLCs [2, 3]. In this study, downregulating MCRS1 expression significantly inhibited the growth of tumor cells *in vitro* and in an animal model.

Next, we clarified the mechanisms by which MCRS1 silencing inhibited tumor proliferation through comparing the mRNA expression profiles of cultured lung cancer cells with or without MCRS1 silencing. The products of the differentially expressed genes directly related to cellular growth were grouped into the following families: the JAK-STAT signaling pathway, the MAPK signaling pathway, the mTOR signaling pathway, the p53 signaling pathway, the pRb1/E2F cell-cycle pathway, and the ErbB signaling pathway. Among the components of these pathways with altered gene expression, several molecules associated with cellular proliferation are discussed below. 1) The expression of two classical TSG (P53 and Rb1) was upregulated at the mRNA level in NSCLC cells with knocked-down MCRS1 expression. These two genes have been universally accepted as playing important roles in preventing the initiation and progression of tumors [26–28]. In previous studies, downregulating MCRS1 expression was found to increase the levels of Rb1 and P21 expression in colorectal cancer cells [16], whereas the ectopic expression of MCRS1 in normal human cells resulted in elevated protein levels of P53 and P21 [22]. 2) The level of expression of the MYC gene was noticeably downregulated following MCRS1 silencing. MYC, which has been long considered to be an oncogene, contributes to oncogenesis through overstimulating cellular growth and metabolism and cell cycle progression [29]. Although the bioinformatic predictions indicated that the MYC gene was potentially regulated by the MCRS1 protein complex, chromatin immunoprecipitation (ChIP) assays revealed that MCRS1 and its complex did not directly bind to MYC (Additional file 7). How MCRS1 affects MYC expression requires further investigation. 3) The levels of vital cell cycle-related molecules (CDK4, CDK6, cyclin A2 and cyclin D1) were remarkably altered in the MCRS1 knockdown NSCLC cells, consistent with previous observations that MCRS1 modulated cellular proliferation through affecting cell cycle-related molecules [9, 16, 21, 22]. 4) The level of E2F2, which plays critical roles in cell cycle regulation and promoting cellular growth [30], was reduced in lung cancer cells with knocked-down MCRS1 levels. 5) As expected, the levels of PCNA and Ki67, which are proliferation markers in many types of human cancer, were decreased in NSCLC cells after MCRS1 silencing. These results suggested that MCRS1 promotes cellular growth through signaling pathways in which these molecules participate. Therefore, we investigated the relationship between the levels of MCRS1 and these signaling molecules in NSCLC. However, the results of the bioinformatic analyses and the ChIP assays showed that MCRS1 did not bind to all of the genes encoding the molecules mentioned above, indicating that MCRS1 may indirectly regulate the expression of these genes through factors such as miRNAs.

Compelling evidence has demonstrated that miRNAs are important regulators of gene transcription [31, 32]. Altered expressions of miRNAs are common events in lung cancer [33]. Our previous study showed that MCRS1 binds to the promoter region of the miR-155 RNA host gene (MIR155HG) and upregulates the expression of miR-155 [3]. MiR-155 has been reported to act as an oncogenic miRNA by promoting tumor growth, the EMT process and invasion/metastasis [34–38]. Thus, we sought to investigate whether MCRS1 promoted tumor growth through miR-155-targeted genes. Firstly, the bioinformatic analyses demonstrated that miR-155 can bind to Rb1 mRNA. Secondly, the level of Rb1 protein expression was inversely correlated with that of miR-155 in lung cancer tissues, which was consistent with the principle of the miRNA-mediated regulation of mRNA expression (i.e., the general inverse relationship between the level of expression of a miRNA and those of its targets). Thirdly, we transfected miR-155 mimics or inhibitors into NSCLC cells and found that the forced expression of miR-155 reduced Rb1 levels and vice versa. Fourthly, the results of the luciferase reporter assays revealed that miR-155 bound to the 3′UTR of Rb1. Finally, a cellular functional assay demonstrated that Rb1 overexpression partially abrogated the positive effect of miR-155 on the growth of NSCLC cells. Rb1 has been reported to function as a tumor suppressor and to play critical roles in controlling the rate of cellular growth and of tumorigenesis [23, 24]. Rb2, one of Rb family members, is down-regulated in lung cancer [39]. Here, our findings indicated that Rb1 is a functional target of miR-155. Moreover, we confirmed the following facts: 1) that the level of Rb1 protein expression was also reversely correlated with that of MCRS1; 2) that the increase in the level of Rb1 protein caused by MCRS1 knockdown was decreased by upregulating the level of miR-155 in NSCLC cells, whereas decreasing the level of Rb1 protein in the MCRS1 upregulated cells could be increased by downregulating the expression of miR-155. Collectively, these results indicated a new avenue for the growth-promotion effect of MCRS1 on NSCLC cells, which is conducted via the miR-155-Rb1 pathway. Rb1 is one of the most important TSGs known. An Rb1 mutation is associated with the development of certain types of cancer [24]. Moreover, the data obtained in this study indicated that apart from the Rb1 genetic mutation, abnormal levels of MCRS1 and miR-155 also affected the expression and function of Rb1, thereby contributing to carcinogenesis. Our findings provide new insights into the mechanisms regulating Rb1 expression.

Alterations in expressions and functions of oncogenes and TSGs are considered as the major cause of carcinogenesis. The functional synergy of oncogenes and TSGs had been noticed. Interestingly, our study reveals an important fact that there are regulatory relationships between oncogenes and TSGs apart from the functional synergy. Furthermore, we found that miRNA is one of mediators between oncogene and TSG. In another research, authors found that there is p53-miR-200-ZEB1/BMI1 pathway and that the pathway regulates EMT and stemness properties in breast cancer cells [40]. Thus, we put forward the hypothesis that there are regulatory relationships between oncogenes and TSGs; the oncogene-miRNA-TSG networks are one of mechanisms among the regulatory relationships; the regulatory relationships and the networks might play active roles in the development and progression of cancer. Alterations in the gene expression are involved in complex mechanisms including genetic mutation, DNA copy number, transcription factor, DNA methylation, histone modification, non-coding RNA etc. The abnormal expressions of some oncogenes and TSGs in certain tumors have been elucidated, however, the most mechanisms underlying the abnormal expressions of oncogenes and TSGs in cancers remains elusive. For examples, genetic mutations can lead to loss or gain of gene functions, but loss or gain of gene functions in some cancers are not caused by genetic mutations, even their reasons are unclear up to now. We speculate that the oncogene-miRNA-TSG network may be an important regulator of the expressions of oncogenes and TSGs in cancers apart from the classical mechanisms.

MCRS1 overexpression has been observed in human tumors. In a previous study, we demonstrated that MCRS1 overexpression resulted partially from the downregulation of the level of miR-129* in NSCLCs [3]. However, the molecular mechanisms underlying the alteration in MCRS1 expression were not entirely clear. Many studies have shown that gene copy variation directly affected gene expression; for example, the DNA copy number of HER-2 and EGFR mediated the overexpression of these genes in breast and lung cancer, respectively [41, 42]. In this study, we analyzed the MCRS1 DNA copy number and found that alterations in the MCRS1 DNA copy number were consistent with the levels of MCRS1 mRNA and protein observed; the MCRS1 DNA copy number in NSCLC tissues and cultured lung cancer cells was dramatically elevated compared with that in normal cells. We believe that the MCRS1 DNA copy number affected the expression of the MCRS1 gene to some extent. Additionally, we analyzed the results we obtained using whole-genome sequencing of 14 cases of NSCLC in another study [43] and published the datasets obtained using NSCLC whole-genome sequencing [44, 45], and we did not find any MCRS1 gene mutations in the NSCLC samples. Accordingly, changes in the MCRS1 DNA copy number may be a mechanism underlying its overexpression in NSCLC cells, but mutation of this gene is not necessary for its abnormal regulation. Moreover, considering that the relationships between the levels of MCRS1 and miR-155 as well as those of miR-155 and Rb1, we explored whether an alteration in the MCRS1 DNA copy number affected the expression of Rb1 and miR-155. Although there were correlations between the MCRS1 DNA copy number and the level of MCRS1 expression as well as between the levels of MCRS1 and miR-155 expression, our data did not demonstrate correlations between the MCRS1 DNA copy number and the levels of Rb1 or miR-155 expression (Additional file 8). These data indicated that factors other than changes in the MCRS1 DNA copy number also regulated MCRS1 expression in NSCLC cells, e.g., the level of miR-129* expression [3]. Similarly, factors other than the levels of MCRS1 and miR-155 may also affect Rb1 expression. The phenomena observed in this study suggested that the regulatory mechanisms in effect and the relationships between the levels of these molecules are extremely complex in cancer cells and that their regulation does not follow a single linear pattern but may rather involve a network.

## Conclusions

**Fig. 5:**
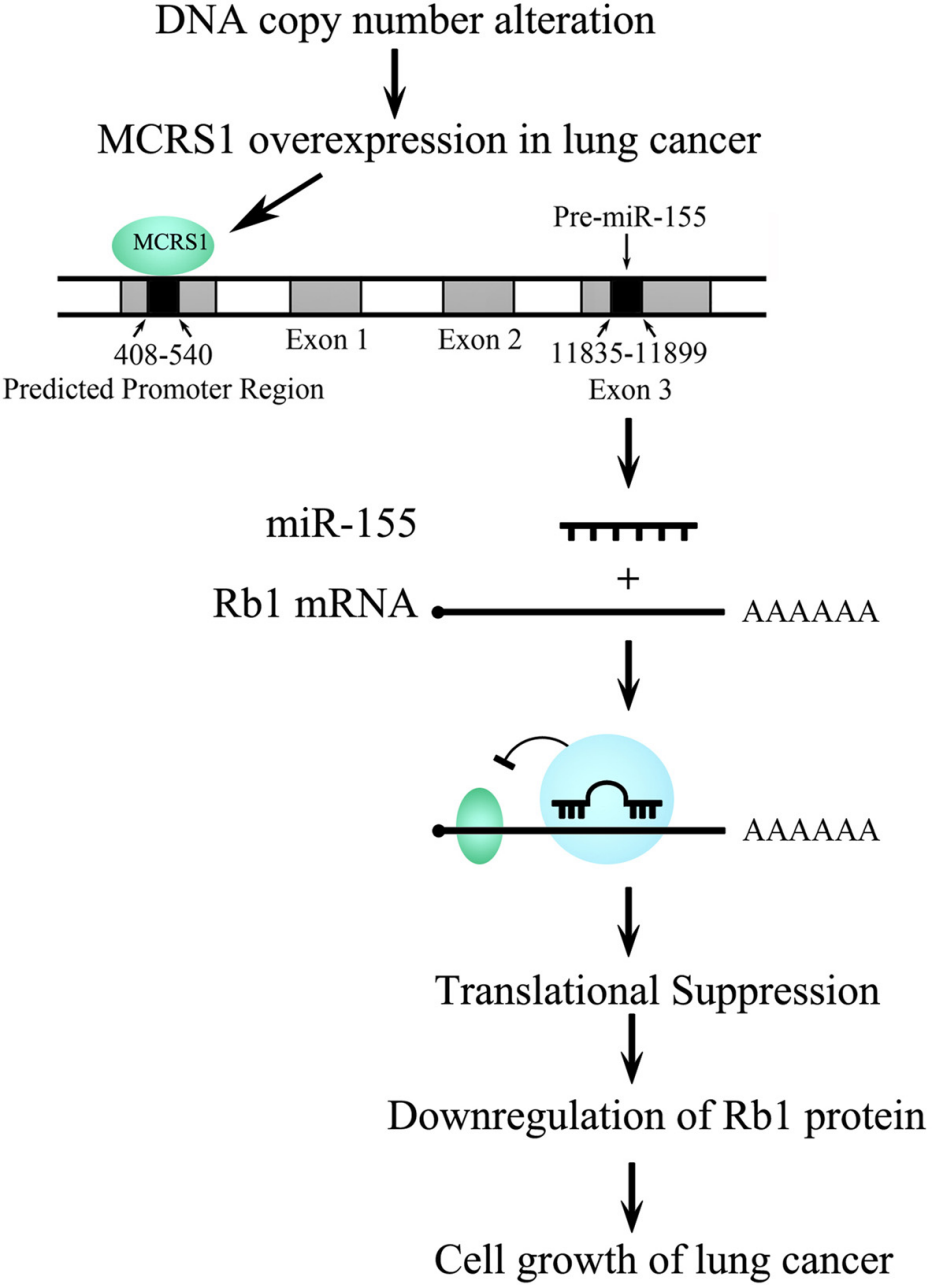
Proposed model for MCRS1-mediated regulation of the proliferation of NSCLC cells

MCRS1 overexpression promoted the proliferation of NSCLC cells and regulated their growth via various pathways. In this study, we clearly demonstrated that MCRS1 regulated the growth of NSCLC cells through the miR-155-Rb1 pathway (Fig. 5). Additionally, MCRS1 overexpression was mediated by alterations in the DNA copy number. MCRS1 is a candidate oncogene, and Rb1 is one of the most important tumor suppression genes. We put forward the hypothesis that there are regulatory relationships between oncogenes and TSGs apart from the functional synergy of both; the oncogene-miRNA-TSG networks are one of mechanisms among the regulatory relationships; the regulatory relationships and the networks might play active roles in the development and progression of cancer. Our study provides new insights into the understanding of relationships between oncogene and TSG as well as abnormal expressions of oncogenes and TSGs in cancers.

## Additional files

Additional file 1:
**Clinical and Pathologic Information of the Patients.** (DOC 61 kb) Additional file 2:
**The cell lines used in this study.** (DOC 16 kb) Additional file 3:
**The primers used in this study.** (DOC 42 kb) Additional file 4:
**The differently expressed genes after MCRS1 silencing in EPLC-32 M1 cells compared with control cells using microarray analysis.** (XLS 129 kb) Additional file 5:
**The results of analyzing the correlation between miR-155 and MCRS1 protein as well as MCRS1 protein and Rb1 protein in NSCLC tissues.** (TIFF 143 kb) Additional file 6:
**The results of analyzing the correlation between MCRS1 protein and its copy number, as well as MCRS1 protein and its mRNA expression in NSCLC tissues.** (TIFF 328 kb) Additional file 7:
**The results of studying the association of the MYC gene and MCRS1 using a chromatin immunoprecipitation (ChIP)-PCR assay.** (DOC 87 kb) Additional file 8:
**The results of analyzing the correlation between the MCRS1 DNA copy number and the levels of expression of MCRS1 mRNA, miR-155, and Rb1 in this study.** (DOC 32 kb)

## Abbreviations

MCRS1: Microspherule protein 1
NSCLC: Non-small cell lung cancer
AC: Adenocarcinoma
SCC: Squamous cell carcinoma
LCC: Large-cell carcinoma
TSG: Tumor suppression gene
MiRNA: microRNA
EMT: Epithelial-mesenchymal transition
QRT-PCR: Quantitative real-time polymerase chain reaction
ChIP assays: Chromatin immunoprecipitation assays
ASO: Antisense oligonucleotides
pRL-TK: *Renilla* luciferase expression plasmid
SDS-PAGE: Sodium dodecylsulfate polyacrylamide gel electrophoresis
SD: Standard deviation
Rb: Retinoblastoma-related gene
3′-UTR: 3′ untranslated region
