# Supplementary material for: The candidate oncogene (MCRS1) promotes the growth of human lung cancer cells via the miR–155–Rb1 pathway
Source: J Exp Clin Cancer Res. 2015 Oct 14;34:121. doi: 10.1186/s13046-015-0235-5 (PMC4606992; doi:10.1186/s13046-015-0235-5)
Supplement: Additional file 1: — Clinical and Pathologic Information of the Patients. (DOC 61 kb) [file 13046_2015_235_MOESM1_ESM.doc]

Additional file 1. Clinical and Pathologic Information of the Patients

| Case no. | Gender | Age | Smoking | Histological type | TNM | Stage |
| --- | --- | --- | --- | --- | --- | --- |
| 1 | Female | 62 | NO | ACA | T2N0M0 | ⅠB |
| 2 | Male | 38 | YES | SCC | T2N3M0 | ⅢB |
| 3 | Female | 42 | NO | ACA | T0M0N0 | ⅠA |
| 4 | Male | 66 | NO | SCC | T2N0M0 | ⅠB |
| 5 | Female | 52 | NO | ACA | T2N0M0 | ⅠB |
| 6 | Female | 41 | NO | ACA | T2N3M0 | ⅢB |
| 7 | Female | 47 | NO | LCC | T3N0M0 | ⅡB |
| 8 | Male | 76 | YES | SCC | T2N0M0 | ⅠB |
| 9 | Male | 57 | YES | ACA | T2aN0M0 | ⅠB |
| 10 | Male | 69 | YES | ACA | T3N0M0 | ⅡB |
| 11 | Male | 38 | NO | ACA | T2N0M0 | ⅠB |
| 12 | Female | 50 | NO | ACA | T2N0M0 | ⅠB |
| 13 | Female | 68 | NO | ACA | T1N0M0 | ⅠA |
| 14 | Male | 60 | YES | SCC | T1N3M0 | ⅢB |
| 15 | Female | 65 | NO | SCC | T3N1M0 | ⅢA |
| 16 | Male | 69 | YES | ACA | T2N0M0 | ⅠB |
| 17 | Female | 62 | NO | ACA | T2N0M0 | ⅠB |
| 18 | Male | 39 | YES | ACA | T1N1M0 | ⅡA |
| 19 | Male | 47 | YES | ACA | T1N0M0 | ⅠA |
| 20 | Female | 62 | YES | ACA | T2N1M0 | ⅡB |
| 21 | Male | 67 | YES | SCC | T1N0M0 | ⅠA |
| 22 | Male | 42 | YES | ACA | T2N1M0 | ⅡB |
| 23 | Male | 70 | YES | SCC | T2N0M0 | ⅠB |
| 24 | Male | 62 | YES | ACA | T1N1M0 | ⅡA |
| 25 | Female | 54 | NO | ACA | T3N0M0 | ⅡB |
| 26 | Female | 37 | NO | ACA | T2N0M0 | ⅠB |
| 27 | Male | 58 | YES | ACA | T3N2M0 | ⅢA |
| 28 | Male | 43 | YES | ACA | T2N1M0 | ⅡB |
| 29 | Male | 39 | NO | SCC | T2N0M0 | ⅠB |
| 30 | Female | 58 | NO | ACA | T2N1M0 | ⅡB |

ACA, adenocarcinomas; SCC, squamous cell carcinomas; LCC, large cell carcinomas.
