# Supplementary material for: The candidate oncogene (MCRS1) promotes the growth of human lung cancer cells via the miR–155–Rb1 pathway
Source: J Exp Clin Cancer Res. 2015 Oct 14;34:121. doi: 10.1186/s13046-015-0235-5 (PMC4606992; doi:10.1186/s13046-015-0235-5)
Supplement: Additional file 2: — The cell lines used in this study. (DOC 16 kb) [file 13046_2015_235_MOESM2_ESM.doc]

**Additional file 2. The cell lines used in this study**

| **Name** | **Cell type** | **Culture media** | **Resource** |
| --- | --- | --- | --- |
| 801D | Large cell lung cancer | RPMI 10%FBS | Cell Bank of Chinese Academy of Science |
| EPLC-32M1 | Lung squamous carcinoma | RPMI 10%FBS | German Cancer Research Center |
| A549 | Lung adenocarcinoma | RPMI 10%FBS | American Type Culture Collection |
| NCI-H292 | Mucoepidermoid lung  carcinoma | RPMI 10%FBS | American Type Culture Collection |
| 16HBE | Immortalized human bronchial epithelial cell | DMEM 10%FBS | Cell Bank of the Peking Union Medical College |
| 293TN | Human embryonic kidney cells | DMEM 10%FBS | American Type Culture Collection |
