# Supplementary material for: The candidate oncogene (MCRS1) promotes the growth of human lung cancer cells via the miR–155–Rb1 pathway
Source: J Exp Clin Cancer Res. 2015 Oct 14;34:121. doi: 10.1186/s13046-015-0235-5 (PMC4606992; doi:10.1186/s13046-015-0235-5)
Supplement: Additional file 3: — The primers used in this study. (DOC 42 kb) [file 13046_2015_235_MOESM3_ESM.doc]

**Additional file 3. The primers used in this study.**

| **Plasmid construction** | **Forward primers (5'-3');**  **Reverse primers (5'-3')** |
| --- | --- |
| **MCRS1 shRNA** | GATCCGCTGAAGAACAACGGTGATTTCAAGAGAATCACCGTTGTTCTTCAGCTTTTTTG;  AATTCAAAAAAGCTGAAGAACAACGGTGATTCTCTTGAAATCACCGTTGTTCTTCAGCG |
| **Rb1 3'UTR** | GGGGTACCGTACACCTCTGGATTCATTGT;  CCGCTCGAGTACACGATCTCTGAAGTTCCT |
| **Rb1 3'UTR mutation** | GCTGTAGGAGAAAAAGAGGCG;  TCTCCTACAGCTGTTTTAATT |
| **Rb1 over-expression vector** | CTAGCTAGCCGTCATGCCGCCCAAAACCC;  ATTTGCGGCCGCCCTCATTTCTCTTCCTTGTTTGA |
| **mRNA expression analysis** | **Forward primers (5'-3');**  **Reverse primers (5'-3')** |
| **MCRS1** | TTGGGCACCATCCCTAAACG；  GGGGCTTTGGATACCTTCTTCTT |
| **MYC** | GTCAAGAGGCGAACACACAAC；  TTGGACGGACAGGATGTATGC |
| **E2F2** | CGTCCCTGAGTTCCCAACC；  GCGAAGTGTCATACCGAGTCTT |
| **Ki67** | GAATGAATGCAGAAATCAGCGGTA; GATCATGGATGACGCTGTGAGAA |
| **PCNA** | ACACTAAGGGCCGAAGATAACG；  ACAGCATCTCCAATATGGCTGA |
| **TP53** | GAGGTTGGCTCTGACTGTACC;  TCCGTCCCAGTAGATTACCAC |
| **Rb1** | CTCTCGTCAGGCTTGAGTTTG;  GACATCTCATCTAGGTCAACTGC |
| **Cyclin A2** | GGATGGTAGTTTTGAGTCACCAC;  CACGAGGATAGCTCTCATACTGT |
| **Cyclin D1** | CAATGACCCCGCACGATTTC;  CATGGAGGGCGGATTGGAA |
| **CDK4** | ATGGCTACCTCTCGATATGAGC；  CATTGGGGACTCTCACACTCT |
| **CDK6** | CCAGATGGCTCTAACCTCAGT；  AACTTCCACGAAAAAGAGGCTT |
| **GAPDH** | TGTTGCCATCAATGACCCCTT;  CTCCACGACGTACTCAGCG |
| **DNA copy number** | **Forward primers ((5'-3');**  **Reverse primers ((5'-3')** |
| **MCRS1 copy number primers** | CACCAGAAGGCTCACTCTTCA;  TGTCTGGTTGTCGAAGTCCGG |

1: For miRNAs expression analysis, forward primers of miR-155 was purchased from Guangzhou RiboBio Co., Ltd；U6 and miRNA reverse primer were purchased from Qiagen, Hilden, Germany.

2: For DNA copy number analysis, Multicopy reference (MRef) was purchased from Qiagen.
