# Supplementary figures and images for: The candidate oncogene (MCRS1) promotes the growth of human lung cancer cells via the miR–155–Rb1 pathway
Source: J Exp Clin Cancer Res. 2015 Oct 14;34:121. doi: 10.1186/s13046-015-0235-5 (PMC4606992; doi:10.1186/s13046-015-0235-5)

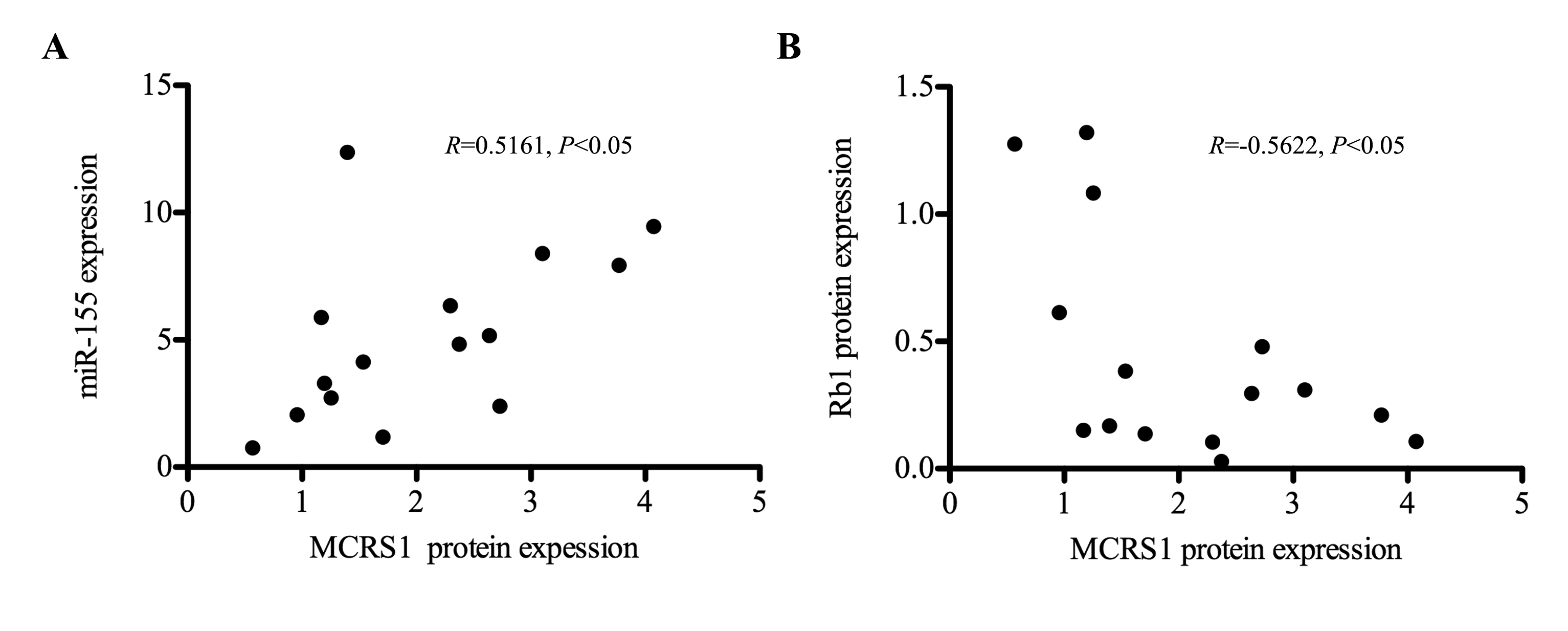

Supplement: Additional file 5: — The results of analyzing the correlation between miR-155 and MCRS1 protein as well as MCRS1 protein and Rb1 protein in NSCLC tissues. (TIFF 143 kb) [file 13046_2015_235_MOESM5_ESM.tif]

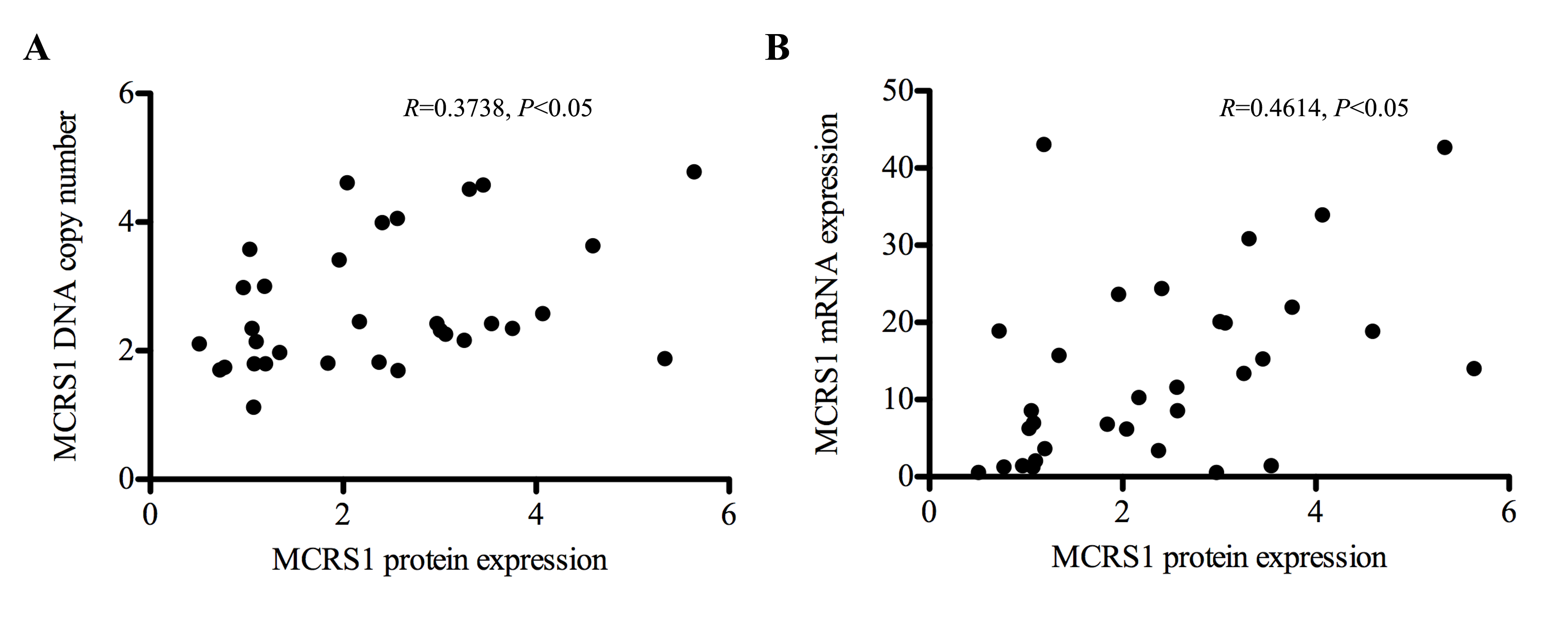

Supplement: Additional file 6: — The results of analyzing the correlation between MCRS1 protein and its copy number, as well as MCRS1 protein and its mRNA expression in NSCLC tissues. (TIFF 328 kb) [file 13046_2015_235_MOESM6_ESM.tif]
