# Supplementary material for: The candidate oncogene (MCRS1) promotes the growth of human lung cancer cells via the miR–155–Rb1 pathway
Source: J Exp Clin Cancer Res. 2015 Oct 14;34:121. doi: 10.1186/s13046-015-0235-5 (PMC4606992; doi:10.1186/s13046-015-0235-5)
Supplement: Additional file 7: — The results of studying the association of the MYC gene and MCRS1 using a chromatin immunoprecipitation (ChIP)-PCR assay. (DOC 87 kb) [file 13046_2015_235_MOESM7_ESM.doc]

**Additional file 7.** Studying relationships between MYC and MCRS1 through chromatin immunoprecipitaion (ChIP)-PCR assay

UniProt website ([http://www.uniprot.org](http://www.uniprot.org/)) and TFBID software (<http://tfbind.hgc.jp/>) were respectively used to search for MCRS1 complexes and to predict binding sites of MCRS1 complexes. The promoter region of MYC was determined through Transcriptional Regulatory Element Database (TRED) (<https://cb.utdallas.edu/cgi-bin/TRED/tred.cgi?process=home>). Then we designed the paired primers and performed ChIP-PCR assay to investigate whether MCRS1 could bind to the promoter region of MYC. The primers and results were demonstrated as follow:

| **Promoter region of MYC** | **Forward primers (5'-3');**  **Reverse primers (5'-3')** | **Results of ChIP-PCR** |
| --- | --- | --- |
| **MYC （34-149）** | AATACATGACTCCCCCCAAC;  AGGAGGAAAACGATGCCTAG | Negative |
| **MYC （137-253）** | TCGTTTTCCTCCTTATGCCT;  TTTGATCAAGAGTCCCAGGG | Negative |
| **MYC （237-374）** | TGGGACTCTTGATCAAAGCG;  TATAAATCATCGCAGGCGGA | Negative |
| **MYC （374-494）** | ACTCACAGGACAAGGATGCG;  GCCGCATGAATTAACTACGC | Negative |
| **MYC （489-638）** | TGCGGCTCTCTTACTCTGTT;  TCTTTTTTCTTTTCCCCCAC | Negative |
| **MYC （618-685）** | CGTGGGGGAAAAGAAAAAAG;  ACCCTCGCATTATAAAGGGC | Negative |
