# Supplementary material for: The candidate oncogene (MCRS1) promotes the growth of human lung cancer cells via the miR–155–Rb1 pathway
Source: J Exp Clin Cancer Res. 2015 Oct 14;34:121. doi: 10.1186/s13046-015-0235-5 (PMC4606992; doi:10.1186/s13046-015-0235-5)
Supplement: Additional file 8: — The results of analyzing the correlation between the MCRS1 DNA copy number and the levels of expression of MCRS1 mRNA, miR-155, and Rb1 in this study. (DOC 32 kb) [file 13046_2015_235_MOESM8_ESM.doc]

**Additional file 8.** Analysis of correlation between MCRS1 DNA copy number and expressions of MCRS1 mRNA, miR-155, and Rb1 in this study.

| **Group** | ***R*** | **P value** |
| --- | --- | --- |
| **MCRS1 DCN^1^ and MCRS1mRNA** | 0.4 | * |
| **MCRS1 DCN^1^ and miR-155** | 0.035 | NS |
| **MCRS1 DCN^1^ and Rb1** | -0.269 | NS |
| **MiR-155 and Rb1 protein level** | -0.554 | * |

1. MCRS1 DNA copy number: MCRS1 DCN; NS: No Significance.

2. * P value<0.05
